# Sequence Analysis of *APOA5* Among the Kuwaiti Population Identifies Association of rs2072560, rs2266788, and rs662799 With TG and VLDL Levels

**DOI:** 10.3389/fgene.2018.00112

**Published:** 2018-04-09

**Authors:** Anfal A. Jasim, Suzanne A. Al-Bustan, Wafa Al-Kandari, Ahmad Al-Serri, Huda AlAskar

**Affiliations:** ^1^Department of Biological Sciences, Faculty of Science, Kuwait University, Kuwait, Kuwait; ^2^Human Genetics Unit, Department of Pathology, Faculty of Medicine, Kuwait University, Kuwait, Kuwait

**Keywords:** *APOA*5, re-sequencing, SNPs, genetic association, Kuwait, Arabs

## Abstract

Common variants of Apolipoprotein A5 (*APOA*5) have been associated with lipid levels yet very few studies have reported full sequence data from various ethnic groups. The purpose of this study was to analyse the full *APOA5* gene sequence to identify variants in 100 healthy Kuwaitis of Arab ethnicities and assess their association with variation in lipid levels in a cohort of 733 samples. Sanger method was used in the direct sequencing of the full 3.7 Kb *APOA5* and multiple sequence alignment was used to identify variants. The complete *APOA5* sequence in Kuwaiti Arabs has been deposited in GenBank (KJ401315). A total of 20 reported single nucleotide polymorphisms (SNPs) were identified. Two novel SNPs were also identified: a synonymous 2197G>A polymorphism at genomic position 116661525 and a 3′ UTR 3222 C>T polymorphism at genomic position 116660500 based on human genome assembly GRCh37/hg:19. Five SNPs along with the two novel SNPs were selected for validation in the cohort. Association of those SNPs with lipid levels was tested and minor alleles of three SNPs (rs2072560, rs2266788, and rs662799) were found significantly associated with TG and VLDL levels. This is the first study to report the full *APOA5* sequence and SNPs in an Arab ethnic group. Analysis of the variants identified and comparison to other populations suggests a distinctive genetic component in Arabs. The positive association observed for rs2072560 and rs2266788 with TG and VLDL levels confirms their role in lipid metabolism.

## Introduction

Apolipoprotein gene (*APOA5*) was first described in 2001 as a result of comparative genome sequencing between mouse and man (Pennacchio et al., 2001). It is located on chromosome 11q23 in the APOA1/C3/A4 gene cluster. The human *APOA5* gene usually gives a 366 amino acid long apoA5 protein (Pennacchio et al., 2001) which is predominantly produced in the liver. ApoA5 protein can be found both intracellulary and associated with cell membrane structures (Nilsson et al., 2011). In plasma, apoA5 is present as a monomer on chylomicrons, VLDL and frequently on HDL at extremely low concentration (Nilsson et al., 2011). The specific role of apoA5 has not been yet clearly defined, however, since its discovery to date many studies have been conducted to investigate its effect on lipid metabolism (Weinberg et al., 2003; Olofsson, 2005; Beckstead et al., 2007; Forte et al., 2009; Guardiola et al., 2012).

Several mechanisms have been proposed to explain apoA5 mode of action. Based on such findings, it was concluded that in plasma, apoA5 induced both an increase in lipolysis, through the increase in lipoprotein lipase LPL activity, and an elevated removal of lipoprotein particles, particularly, the triglycerides-rich lipoproteins (TG-RL) (Grosskopf et al., 2005). Also, apoA5 serves as a ligand for an endothelial cell surface protein called glycosylphosphatidylinositol high-density lipoprotein binding protein 1 (GPIHBP1) (Sharma et al., 2012). It was found that GPIHBP1 plays a particular and critical role in the lipolysis processing of chylomicrons promoting LPL lipolytic processing of the TG content of these particles (Beckstead et al., 2003; Beigneux et al., 2007; Sharma et al., 2012). In addition to its important role in modulating plasma TG levels, some experiments have indicated apoa5 effect on reducing HDL-C levels (Beckstead et al., 2003; Grosskopf et al., 2005; Cha et al., 2014). Beside its extracellular effect, previous investigation of apoA5 impact has pointed an action within hepatocytes to modulate hepatic TG metabolism and secretion (Weinberg et al., 2003; Olofsson, 2005). In hepatocytes, apoA5 was found responsible for the attenuation of the second-step of VLDL particle maturation, essential for the formation of TG-rich VLDL and therefore impairing liver VLDL assembly and secretion (Walther and Farese, 2012).

However, recent advances in genome-wide association studies (GWAS) have provided a fairly good view of the contribution of *APOA5* variants to dyslipidemia in several populations (Talmud, 2007; Li et al., 2014; Zubair et al., 2016; Kefi et al., 2017; Sumegi et al., 2017). The minor allele of several common *APOA5* SNPs such as rs662799 (-1131T>C) and rs3135506 (S19W) have been reported to be consistently and significantly associated with plasma triglyceride (TG) levels (Baum et al., 2003; Chaaba et al., 2005) and the risk of cardiovascular disease (Talmud et al., 2002; Lai et al., 2004) in several populations. In addition to the well characterized common *APOA5* variants, rare variants in the *APOA5* gene have been documented and implicated in the development of dyslipidemia and associated diseases (Priore Oliva et al., 2006; Ouatou et al., 2014). But while that disease etiology is common between populations, risk variants can often be population-specific. Therefore, these variants and others have been identified and genotyped in several populations by various molecular methods and the results have not been always been consistent among different ethnicities (Pullinger et al., 2008; Cha et al., 2014; Li et al., 2014; Ouatou et al., 2014; Sumegi et al., 2017).

Few studies have provided full sequence analysis of the APOA5 gene locus and limited studies have been conducted on APOA5 variants and their possible associations among Arab countries (Chaaba et al., 2005; Ouatou et al., 2014; Kefi et al., 2017).

Kuwait is a small country that is situated on the north-east of the Arabian Peninsula and at the northern end of the Arabian Gulf. Historically, the population of Kuwait comprises early settlers originating from the tribes of neighboring Arabian and Persian countries (particularly from Saudi Arabia, Iraq and Iran) and from the nomadic Arabs of the desert, living on the fringes of the Arabian Peninsula, called Bedouins (Teebi, 1994; Al-Bustan et al., 2005; Casey, 2007). The prevalence of disorders such as type 2 diabetes, obesity, hypertension and dyslipidemia has increased (Alwan and King, 1995; AlMajed et al., 2011; Alhyas et al., 2012; Channanath et al., 2013). In 2013, (WHO) estimates that dyslipidemia is showing alarmingly high prevalence and emerging as a risk factor for coronary disease in Kuwait (World Health Organization, 2013). While the settlements and the subsequent admixtures have shaped the genetics of Kuwait, understanding the genetic structure of Kuwaiti population remains a big challenge but necessary to aid studies in defining genetic and molecular mechanisms underlying causes of dyslipidemia and other complex diseases.

Therefore, the main objective of this study was to re-sequence the full *APOA5* gene loci in Kuwaiti Arabs to identify potential variants that could be associated with variation in serum lipid levels and to validate a selection of the variants in a cohort. This study is the first to report full sequence data and analysis at the *APOA5* gene loci in an Arabic ethnic group.

## Materials and methods

### Studied samples

This study was undertaken within the guidelines set by the Declaration of Helsinki, and the protocol was approved by the Local Ethical Committee at Kuwait University. One hundred apparently healthy Kuwaiti Arabs (50 males and 50 females) were recruited from volunteers who attend the regional polyclinics or the major hospitals in Kuwait. Written informed consent was obtained from all subjects. The age range of the subjects was 18–69 years, and the body mass index (BMI; in kg/m2) range was 19–30. Medical and family medical history of hypertension, hypercholesterolemia, hyperglyceridemia, diabetes and cardiovascular diseases were documented. Ethnicity was verified by tracing both maternal and paternal lineages at least four generations using pedigree analysis. The selected subjects were divided into three groups according to their Cholesterol (TC) levels. There were 20 subjects with high TC levels (5.2–6.77 mmol/L), 20 with low TC levels (2.48–3.9 mmol/L) and a remaining 60 with normal TC levels (TC: 4–5.16 mmol/L). Association of identified and selected variants were analyzed in a cohort of 733 samples of the Kuwaiti General population. The cohort included randomized samples of the Kuwaiti general population with documented lipid profiles recruited at various hospitals during routine check-up whose informed consent was obtained individually following the ethical guidelines stated earlier. The sample consisted of 425 females and 279 males with ages ranging between 18 to 76. For each sample, phenotypic variables including BMI and family history of hypercholesterolemia, hypertension, diabetes and heart diseases were recorded. Relevant phonetic data was documented for each sample. A summary of the sample description is provided in Table 1.

**Table 1:** Demographic and clinical features of study cohort (*n* = 733).

**Parameters**	
Sex (Males, Females)	39.63%, 60.37%
Age (yr)	32.53 ± 0.53
BMI	27.19 ± 0.30
Cholesterol (mmol/L)	4.72 ± 0.04
Triglyceride (mmol/L)	0.82 ± 0.78
VLDL (mmol/L)	0.34 ± 0.33
HDL (mmol/L)	1.14 ± 0.01
LDL (mmol/L)	3.00 ± 1.1

### Biochemical measurements

Blood samples were taken intravenously after an overnight fast. Serum lipid levels in each sample were determined by enzymatic methods with commercially available kits and performed on a UniCel DxC 800 Synchron Clinical System from Beckman Coulter (USA) in the Clinical Chemistry facility at Al-Amiri Hospital in Kuwait.

### DNA analysis

Total genomic DNA was extracted from whole blood using salting-out procedure described by Miller et al. (1988). A region of 3.7 Kb of *APOA5* gene was amplified from each individual in eight overlapping segments using newly designed primer sets (*n* = 8 pairs) using Primer3 Input software version 0.4.0 (//Frodo.wi.mit.edu/) (Supplementary Table 1). Amplification reactions were performed by PCR in an Applied Biosystems Fast thermal cycler (Version 1.01, Life Technologies, USA). PCR products were purified using Nucleospin® extract II column Kit (Clontech Laboratories, Inc., Version No. PR48598) following the suggested protocol (Macherey-Nagel, Germany). Cycle sequencing was then performed according to the manufacturer's instructions using the BigDye Terminator v.3.1 in a Fast Thermal Cycler (Life Technologies, Applied Biosystems, USA). The extension products were purified using BigDye® XTerminator™ Kit (Life Technologies, Applied Biosystems, USA). Capillary electrophoresis was then performed in ABI-3130xl Genetic Analyzer (Life Technologies, Applied Biosystems, USA).

### Sequence analysis and polymorphisms identification

The obtained DNA sequences for each sample were analyzed using the AB DNA Sequencing Analysis Software version 5.3.1 (Life Technologies, Applied Biosystems, USA). The sequences from the two reactions were compared for quality assurance and were aligned using ClustalW software (Multiple Alignment Tool) [37] to screen for all novel and/or mutations. Multiple sequence alignment among all samples and in comparison, to the reference sequence (NT_033899.8) was performed in order to confirm the genotype of each sample and to determine the presence of “common,” “rare,” and “novel” SNPs. Information about reported SNPs were obtained from NCBI and Ensembl databases (http://www.ncbi.nlm.nih.gov/, http://www.ensembl.org/).

### Validation of novel SNPs and association of common SNPs identified

A total of 7 SNPs, including the two novels were validated a cohort of 736, with the exception of rs662799 which was only validated in 549 samples of the cohort due to technical difficulties and limited resources. The variants were analyzed using real-time PCR [ABI 7800HT Realtime PCR (GS01/02)] with customized primer and probe sets for the two novel SNP's and commercially available primer and probe sets for the four remaining SNPs (Supplementary Table 2, Applied Biosystems # 4351379). The genotyping assay and protocol was followed based on the manufacturer's recommendations for Taqman™ Genotyping Master Mix (Applied Biosystems # 4371355).

### Haplotype analysis

Linkage disequilibrium (r2) (D′) between the selected SNPs was analyzed using Haploview (version 4.2.) (Barrett et al., 2005). Pairwise r2 and D′ value between each two of SNPs was calculated. An r2 value of 1 means that the two loci are in complete linkage disequilibrium, in contrast an r2 value of 0 means that the two loci are in linkage equilibrium. In this study, r2 values more than 0.7 was considered to signify that two loci are in linkage disequilibrium. Haploview (version 4.2.) was also used to make graphical representations of LD. Haplotypes blocks were defined according to Gabriel et al. definition (Gabriel et al., 2002).

### Statistical analysis

Allele and genotype frequencies for each variant (novel, rare and common) were estimated by simple gene counting method for both the sequenced samples (*n* = 100: 22 variants) and for the cohort (*n* = 733: 6 variants). Hardy-Weinberg equilibrium (HWE) was also tested using the GENEPOP software (Version 4.2) (Rousset, 2008). Statistical analyses on the differences in the genotypic and allele frequency distribution were determined with regards to age, gender and BMI using Kruskal-Wallis ANOVA test and were reported as mean ± standard error. A two-tailed *p*-value of 0.05 was considered statistically significant. TG, HDL-C, LDL-C, VLDL were natural log-transformed to achieve approximate normal distribution before further analysis. In addition, Logistic regression analyses was preformed to investigate the association of the studied SNPs with lipid level variations. Genetic modeling of the significant variants was also performed. All these tests were performed using the R software (version 3.3.1) utilizing the following packages SNPassoc, psych, genetics, and MASS (R Core Team, 2016). Person's Chi-square test was also used to investigate the significant of the different distribution of haplotypes frequencies between the high and normal TG patient's groups. For all tests, the statistical significance level was set at *p* < 0.05.

## Results

### Genetic variants

The full *APOA5* gene including the promoter region, exons and introns, downstream and upstream regions was successfully sequenced for all 100 Kuwaiti Arab samples. The revealed *APOA5* sequence with all of the detected SNPs was deposited in the NCBI GenBank under accession number **KJ401315**. Twenty-two genetic variants, two of which are novel, were observed by scanning the resulted chromatograms (Supplementary Figure 1) as well as by aligning the sequences with a reference *APOA5* sequence **(NT_033899.8)**. The majority of the identified SNPs were in the non-coding region in which; five were intragenic, seven in the 3′ UTR, one in the 5′ UTR, two in the downstream region and two in the upstream region. Further, three missense mutations (rs3135506, rs3135507, rs369952307) plus a previously reported synonymous variant (rs12287066) and a novel synonymous variant in exon 4 (2197G>A) were reported (Table 2). Each novel variant was identified in a heterozygous state in two different individuals. The novel synonymous SNP 2197G>A (at genomic position 116661525; GRCh37/hg:19) causes a G to A transition at position. The individual carrying this variant is a 27 years old female that has a high (28 kg/m^2^), low (0.89 mmol/L) and high (3.52 mmol/L). The second novel variant found in the 3′UTR (at genomic position 116660500; GRCh37/hg:19) results from a C to T transition at position 3,222 belongs to a 20 years old female that has a normal BMI (20.7 kg/m^2^) as well as a normal lipid profile. Functional prediction analysis employing SIFT, TargetScan, SNPNexus tools revealed that the two novel SNP's are simple sequence variants.

**Table 2:** Characteristics of the 22 identified SNPs by re-sequencing the *APOA5* gene locus in Kuwaiti Arabs (*n* = 100).

**SNP Ref. no**.	**Position on submitted sequence**	**Chromosomal position**	**Variant Type**	**Minor allele frequency**
rs1729411^**^	g.4462C>T	g.116792959G>A	Upstream	0.097
rs34003087^**^	g.4541G>A	g.116792880C>T	Upstream	0.051^*^
rs648450	g. 937C>T	g.116662785G>A	Intron	0.005^*^
**rs651821**^∧^	**g. 1143G**>**A**	**g.116661525C**>**T**	**5**′ **UTR**	**0.095**
rs367787801	g. 1273G>C	g.116662449C>G	Intron	0.015^*^
rs41416350	g. 1281G>A	g.116662441C>T	Intron	0.005^*^
rs3135506	g. 1315C>G	g.116662407G>C	Missense (p.Ser19Trp)	0.055^*^
rs12287066	g. 1391C>A	g.116662331G>T	Synonymous (p.Ile44=)	0.13
rs36077557	g. 1687delC	g.116662034delG	Intron	0.31
**rs2072560**	**g. 1896A**>**G**	**g.116661826T**>**C**	**Intron**	**0.08**
**Novel 1**	**g. 2197G**>**A**	**g.116662127C**>**T**	**Synonymous (p.Val140** = **)**	0.005^*^
rs3135507^**^	**g. 2234G**>**A**	**g.116661488C**>**T**	**Missense (p.Val153Met)**	0.045^*^
rs369952307	g. 2813G>A	g.116660909C>T	Missense (p.Asp346Asn)	0.005^*^
rs619054	g. 2909C>T	g.116660813G>A	3′ UTR	0.26
rs192708363	g. 2937C>A	g.116660785G>T	3′ UTR	0.005^*^
rs34089864^**^	g. 2954C>T	g.116660768G>A	3′ UTR	0.015^*^
**rs2266788**	**g. 3036C**>**T**	**g.116660686G**>**A**	**3**′ **UTR**	0.085^*^
rs148759216^**^	g. 3167insAG	g.116660554_116660555insCT	3′ UTR	0.004^*^
**Novel 2**	**g. 3222C**>**T**	**g.116660500G**>**A**	**3**′ **UTR**	0.005^*^
rs33984246^**^	g. 3272T>C	g.116660450A>G	3′ UTR	0.04^*^
rs115102021	g. 3650C>T	g.116660072G>A	Downstream	0.005^*^
rs142958146^**^	g. 3714T>C	g.116660008A>G	Downstream	0.04^*^

*- Chromosomal position is based on chromosome 11 published reference sequence (NC_000011.10) in the NCBI GeneBank database (based on genome assembly GRCh37/hg:19.- Location on the gene is based on the APOA5 published reference sequence (NT_033899.8) in the GeneBank data base*.

* *Only heterozygotes identified with the rare allele*.

** *MAF >0.05 reported in the 1,000 genome project and Gene Bank for other populations. All frequencies were found in HWE (p > 0.05) except for rs36077557. The highlighted are the variants further analyzed for association with lipid levels*.

### Genotype and allele frequencies

All variants were in HWE. Nine variants were common between Kuwaiti Arabs with a minor allele frequency (MAF) above 0.05. The other remaining variants (*n* = 13) were identified as rare with a minor allele frequency < 0.05 including the two novel SNPs (Table 2).

Four of the detected variants (rs2266788, rs651821, rs2072560, and rs3135506) plus the commonly studied rs662799 (−1,131 T>C) SNP that had previously shown to be associated with lipid levels were selected for further analysis to determine their association with variation in serum lipid levels among a Kuwait cohort. The two novel variants were also selected for the validation and further statistical analysis.

### Validation and association of selected variants with serum lipid levels

Real-time PCR was used to validate and genotype a cohort of 736 samples of the general Kuwaiti population. A sample of the real-time PCR allelic discrimination plot for the novel variants using the newly designed primers and probes sets are provided in the Supplementary Figures 2A–F. A summary of the genotype and allele frequencies determined in the 736 samples of the Kuwaiti cohort are provided in Table 3. Both novel variants were only identified in a heterozygous state in < 1 % of the cohort (Table 3) in which Novel 2 was only detected in the original sequenced sample while Novel 1 was further identified in an additional three samples implicating them as “private” variants.

**Table 3:** Genotypic and allelic frequencies of the selected 7 *APOA*5 variants in the cohort of Kuwaiti Arabs (*n* = 733).

**SNPs**	**Genotype**	**Genotype frequency**	***n***	**Minor allele frequency**
**rs651821**	AA	0.75	552	0.13
g. 1143G>A	AG	0.23	166	
c.-3A>G	GG	0.2	15	
**rs2072560**	AA	0.2	10	0.89
g. 1896A>G	AG	0.19	141	
IVS3-476G>A	GG	0.79	582	
715 G>T				
**ra3135506**	CC	0.876	642	0.06
g. 1315C>G	CT	0.121	89	
c.56C>G	TT	0.003	2	
**rs2266788**	CC	0.2	12	0.13
g. 3036C>T	CT	0.22	165	
c.1259T>C	TT	0.76	556	
1891 T>C, c.158C>T				
**Novel SNP 1**	AA	0%	0	0.01
g. 2197G>A	AG	0.005	4	
		GG	0.995	729
**Novel SNP 2**	CC	0.999	732	0.01
g. 3222C>T	CT	0.001	1	
rs662799^*^	CCTT	0.6804	372	C = 0.82
−1131 T>C	CT	0.28	157	0.18
	TTCC	0.0468	20	

* *This variant was validated in only 549 samples*.

The association between the genotypes of the seven variants with variation in serum lipid levels are summarized in Table 4. SNPs rs2072560 and rs2266788 showed a significant association with TG reporting *p*-values of 0.04 and 0.02 respectively. Individuals carrying the minor A allele of rs2072560 (*n* = 107) displayed higher TG levels (>0.98 mmol/L) than individuals (0.92 mmol/L ± 0.04) with the GG genotype (*n* = 404). Carriers of the C allele of rs2266788 (*n* = 125) also displayed higher TG (>0.99 mmol/L) than individuals (0.92 mmol/L ± 0.04) with the TT genotype (*n* = 386). SNPs rs2072560 and rs2266788 also showed significant association with VLDL in which carriers of the A allele of rs2072560 (*n* = 106) showed increased VLDL levels (>0.41 mmol/L) than those (0.39 mmol/L ± 0.02) having the GG genotype (*n* = 397). Also, individuals with the C allele of rs2266788 (*n* = 123) showed higher levels of VLDL (>0.42 mmol/L) than the TT genotype (0.39 mmol/L ± 0.02; *n* = 380). Significant association (*p* = 0.01) of the minor C allele of rs662799 was observed with lower levels of TG (−0.46 mmol/l) as well as lower VLDL levels (−1.29 mmol/L, *p* = 0.03) in the 459 samples analyzed of the cohort (*n* = 733). On the other hand, the novel SNPs did not reveal any significant association with variation in lipid levels.

**Table 4:** Association of the 7 variants at the *APOA5* gene locus with lipid profiles in the Kuwaiti Cohort (*n* = 733).

**SNP**	**Lipid parameter**		**W/W**		**W/M**		**M/M**	***p*-value**
		**n**		**n**		**n**		
rs651821	BMI	386	27.15 ± 0.34	113	27.23 ± 0.56	12	26.92 ± 2.07	0.99
	TC	385	4.89 ± 0.05	113	4.63 ± 0.08	12	4.97 ± 0.27	0.4
	TG	386	0.92 ± 0.04	113	0.97 ± 0.06	12	1.10 ± 0.18	0.15
	VLDL	379	0.39 ± 0.02	112	0.41 ± 0.03	12	0.46 ± 0.08	0.18
	HDL	372	1.2 ± 0.02	109	1.72 ± 0.03	11	1.16 ± 0.07	0.92
	LDL	371	3.08 ± 0.04	109	3.06 ± 0.07	11	3.42 ± 0.29	0.4
rs2072560	BMI	404	26.94 ± 0.34	97	26.94 ± 0.58	10	24.80 ± 1.33	0.68
	TC	403	4.67 ± 0.05	97	4.69 ± 0.09	10	5 ± 0.31	0.28
	TG	404	0.92 ± 0.04	97	0.98 ± 0.06	10	1.10 ± 0.22	0.04^*^
	VLDL	397	0.39 ± 0.02	96	0.41 ± 0.03	10	0.46 ± 0.10	0.04^*^
	HDL	389	1.19 ± 0.02	94	1.18 ± 0.04	9	1.15 ± 0.08	0.93
	LDL	389	3.07 ± 0.04	94	3.09 ± 0.07	9	3.46 ± 0.35	0.25
rs3135506	BMI	447	27.05 ± 0.30	64	27.89 ± 0.93	–	–	0.39
	TC	447	4.66 ± 0.04	63	4.81 ± 0.12	–	–	0.31
	TG	447	0.94 ± 0.03	64	0.93 ± 0.07	–	–	0.99
	VLDL	442	0.40 ± 0.02	61	0.38 ± 0.03	–	–	0.83
	HDL	431	1.18 ± 0.02	61	1.23 ± 0.06	–	–	0.34
	LDL	431	3.07 ± 0.04	61	3.16 ± 0.12	–	–	0.69
rs2266788	BMI	386	27.27 ± 0.34	113	26.98 ± 0.57	12	25.08 ± 1.13	0.77
	TC	385	4.65 ± 0.05	113	4.77 ± 0.08	12	4.78 ± 0.26	0.24
	TG	386	0.92 ± 0.04	113	1.00 ± 0.06	12	0.99 ± 0.20	0.02^*^
	VLDL	380	0.39 ± 0.02	111	0.42 ± 0.02	12	0.42 ± 0.09	0.03^*^
	HDL	371	1.19 ± 0.02	110	1.20 ± 0.04	11	1.18 ± 0.07	0.87
	LDL	371	13.06 ± 0.04	110	3.13 ± 0.07	11	3.22 ± 0.34	0.38
Novel SNP 1	BMI	509	27.17 ± 0.29	2	25.35 ± 4.65	–	–	0.94
	TC	509	27.17 ± 0.29	2	25.35 ± 4.65	–	–	0.75
	TG	509	0.94 ± 0.03	2	0.64 ± 0.26	–	–	0.98
	VLDL	501	0.40 ± 0.01	2	0.26 ± 0.11	–	–	0.93
	HDL	490	1.19 ± 0.02	2	1.21 ± 0.09	–	–	0.68
	LDL	490	3.08 ± 0.04	2	2.80 ± 0.60	–	–	0.83
Novel SNP 2	BMI	510	27.16 ± 0.29	1	28 ± 0.00	–	–	0.83
	TC	509	4.68 ± 0.04	1	4.66 ± 0.00	–	–	0.99
	TG	510	0.94 ± 0.03	1	0.56 ± 0.00	–	–	0.68
	VLDL	502	0.40 ± 0.01	1	0.25 ± 0.00	–	–	0.81
	HDL	491	1.2 ± 0.02	1	0.89 ± 0.00	–	–	0.2
	LDL	491	3.08 ± 0.04	1	3.52 ± 0.00	–	–	0.48
rs662799^**^	BMI	347	26.86 ± 0.33	140	27.80 ± 0.64	19	28.36 ± 1.18	0.26
	TC	347	4.69 ± 0.05	140	4.61 ± 0.08	19	4.60 ± 0.22	0.32
	TG	347	0.97 ± 0.04	140	0.87 ± 0.05	19	0.79 ± 0.15	0.01^*^
	VLDL	346	0.41 ± 0.02	138	0.37 ± 0.02	18	0.35 ± 0.07	0.03^*^
	HDL	333	1.18 ± 0.02	135	1.22 ± 0.03	18	1.25 ± 0.10	0.15
	LDL	333	3.10 ± 0.04	135	3.00 ± 0.07	18	3.00 ± 0.22	0.12

*− W/W homozygous for common allele, W/M heterozygous for the minor allele, M/M homozygous for minor allele*.

*− Lipid levels data are shown as mean ± SEM for TC: total cholesterol; TG, triglycerides; HDL, High-Density Lipoprotein; LDL, Low-Density lipoprotein; VLDL, Very Low-Density Lipoprotein*.

*− p-values were derived from Kruskal-Wallis ANOVA with adjustment for sex, age and BMI assuming a co-dominant genetic model*.

* *Significant values (p < 0.05) are highlighted*.

** *Analyzed in a sample of 549 as the remaining samples of the cohort were not genotyped*.

### Multivariate analysis and genetic modeling of the associated variants

The rs2072560 SNP was found to be an independent predictive factor for both TG and VLVD when controlling for age, sex and BMI with a similar odds ratio of 1.42 (95% CI: 1.03 –1.95, *p* = 0.03). In addition, rs2266788 SNP was also found to be an independent predictive factor for both TG and VLVD when controlling for age, sex and BMI with an odds ratio of 1.15 (95% CI: 1.03–1.27, *p* = 0.01) and an odds ratio of 1.25 (95% CI: 0.92–1.68, *p* = 0.02) respectively. A significant (*p* = 0.01) decrease of 0.73 OR was also observed for homozygous genotypes of the minor C allele for rs662799 concerning TG serum levels. These results are demonstrated in Table 5.

**Table 5:** Results of multivariate analysis of the *APOA5* rs2072560, rs2266788 and rs662799 with triglycerides (TG) and Very Low-Density Lipoprotein (VLDL) levels in the cohort (*n* = 733).

	**Variable**	**OR**	**95% C.I**.	***p*-value**
**TG**				
(RF allele: G)	rs2072560 G/A	1.09	(0.98–1.22)	0.12
	rs2072560 A/A	1.42	(1.03–1.95)	0.03^*^
	Sex (M)	1.27	(1.16–1.39)	2.11E-07^*^
	Age	1.01	(1.01–1.02)	2.81E-15^*^
	BMI	1.02	(1.02–1.03)	2.44E-11^*^
(RF allele: T)	rs2266788 T/C	1.15	(1.03–1.27)	0.01^*^
	rs2266788 C/C	1.24	(0.93–1.67)	0.13
	Sex (M)	1.28	(1.17–1.40)	1.26E-07^*^
	Age	1.01	(1.01–1.02)	2.23E-15^*^
	BMI	1.02	(1.02–1.03)	2.88E-11^*^
(RF allele: T)	rs662799C/T	0.9	(0.82–1)	0.05
	rs662799T/T	0.73	(0.58–0.92)	0.01^*^
	Sex (M)	1.27	(1.16–1.39)	4.89E-07^*^
	Age	1.02	(1.01–1.02)	4.0E-15^*^
	BMI	1.02	(1.02–1.03)	1.29E-11^*^
**VLDL**				
(RF allele: G)	rs2072560 G/A	1.09	(0.98–1.23)	0.11
	rs2072560 A/A	1.42	(1.03–1.97)	0.03^*^
	Sex (M)	1.27	(1.15–1.39)	1.02E-06^*^
	Age	1.02	(1.01–1.02)	< 2E-16^*^
	BMI	1.02	(1.02–1.03)	2.22E-11^*^
(RF allele: T)	rs2266788 T/C	1.14	(1.02–1.27)	0.02^*^
	rs2266788 C/C	1.25	(0.92–1.68)	0.15
	Sex (M)	1.27	(1.16–1.39)	6.05E-07^*^
	Age	1.02	(1.01–1.02)	< 2E-16^*^
	BMI	1.02	(1.02–1.03)	2.84E-11^*^
(RF allele: C)	rs662799C/T	0.9	(0.81–1)	0.05
	rs662799T/T	0.78	(0.61–1)	0.05
	Sex (M)	1.27	(1.15–1.39)	9.73E-07^*^
	Age	1.02	(1.01–1.02)	<2E-16^*^
	BMI	1.03	(1.02–1.03)	1.12E-11^*^

RF: Reference allele. The values represented include the odds ratio, confidence interval and p values as evaluated by multivariate logistic regression in 511 Kuwaiti individuals. It shows the mentioned variants to be a significant

* *(p < 0.05) independent predictive factor when controlling for age, sex and BMI for both TG and VLDL. Significant values (p < 0.05) are highlighted*.

The genetic modeling of the significant variants was preformed, and results are summarized in Table 6. A significant association of SNP rs2072560 with TG and VLDL was confirmed in which AA genotype shows a 42% increase of having higher TG and VLDL levels regardless of age, sex and BMI as compared to the wildtype GG with an odds ratio of 1.42 (95% CI 1.03–1.95, *p*-value of 0.04) and odds ratio of 1.42 (95% CI 1.03–1.97, *p*-value of 0.04) respectively. Moreover, a significant *p*-value for all modes of inheritance was observed with regards to variant to rs2072560 in association to TG, VLDL except for the over dominant model. Similarly, rs2266788 also revealed genetic association with TG and VLDL regardless of age, sex and BMI. The CC genotype for the this variant showed a 25% increase chance of having high TG and VLDL in comparison with the wild type genotype TT with an odds ratio of 1.25 (95% CI 0.93–1.68, *p*-value of 0.02) and odds ratio of 1.25 (95% CI 0.92–1.68, *p*-value of 0.03) respectively. A significant genetic association for all modes of inheritance was also observed for rs662799 with -TG, VLDL except for the over dominant model.

**Table 6:** Genetic modeling of *APOA5* rs2072560, rs2266788 and rs662799 with TG and VLDL levels in the cohort (*n* = 733).

**Variant association**	**Genetic model**	**n/%**	**OR**	***p*-value**
**rs2072560 relation with TG**	**Codominant**			
	G/G	404 (79.1)	1	**0.04**
	G/A	97 (19)	1.09 (0.98–1.22)	
	A/A	10 (2)	1.42 (1.03–1.95)	
	Dominant			
	G/G	404 (79.1)	1	**0.04**
	G/A-A/A	107 (20.9)	1.12 (1.00–1.25)	
	Recessive			
	G/G-G/A	501 (98)	1	**0.04**
	A/A	10 (2)	1.39 (1.02–1.92)	
	Over-dominant			
	G/G-A/A	414 (81)	1	0.16
	G/A	97 (19)	1.08 (0.97–1.21)	
	log-Additive			
	0,1,2		1.13 (1.02–1.23)	**0.02**
**rs2072560 relation with VLDL**	**Codominant**			
	G/G	397 (78.2)	1	**0.04**
	G/A	96 (19.1)	1.09 (0.98–1.23)	
	A/A	10 (2)	1.42 (1.03–1.97)	
	Dominant			
	G/G	397 (78.2)	1	**0.04**
	G/A-A/A	106 (21.1)	1.13 (1.01–1.26)	
	Recessive			
	G/G-G/A	493 (98)	1	**0.04**
	A/A	10 (2)	1.39 (1.01–1.93)	
	Over-dominant			
	G/G-A/A	407 (80.9)	1	0.15
	G/A	96 (19.1)	1.08 (0.97–1.22)	
	log-Additive			
	0,1,2		1.13 (1.02–1.25)	**0.02**
**rs2266788 relation with TG**	**Codominant**			
	T/T	386 (75.5)	1	**0.02**
	T/C	113 (22.1)	1.15 (1.03–1.27)	
	C/C	12 (2.4)	1.25 (0.93–1.68)	
	Dominant			
	T/T	386	1	**0.01**
	T/C-C/C	125 (24.5)	1.15 (1.04–1.28)	
	Recessive			
	T/T-T/C	499 (97.7)	1	0.2
	C/C	12 (2.4)	1.21 (0.90–1.62)	
	Over-dominant			
	T/T-C/C	398 (77.9)	1	**0.02**
	T/C	113 (22.1)	1.14 (1.02–1.26)	
	log-Additive			
	0,1,2		1.14 (1.04–1.25)	**0.01**
**rs2266788 relation with VLDL**	**Codominant**			
	T/T	380 (75.5)	1	**0.03**
	T/C	111 (22.1)	1.14 (1.02–1.27)	
	C/C	12 (2.4)	1.25 (0.92–1.68)	
	Dominant			
	T/T	380 (75.5)	1	**0.01**
	T/C-C/C	123 (24.5)	1.15 (1.03–1.27)	
	Recessive			
	T/T-T/C	491 (97.6)	1	0.21
	C/C	12 (2.4)	1.21 (0.90–1.63)	
	Over-dominant			
	T/T-C/C	392 (77.9)	1	**0.03**
	T/C	111 (22.1)	1.13 (1.01–1.26)	
	log-Additive			
	0,1,2		1.13 (1.03–1.23)	**0.01**
**rs662799 relation with TG**	**Codominant**			
	C/C	347 (68)	1	**0.01**
	C/T	140 (28)	0.9 (0.82–1)	
	T/T	19 (4)	0.73 (0.58–0.92)	
	Dominant			
	C/C	347	1	**0.01**
	C/T-T/T	159	0.89 (0.80–0.97)	
	Recessive			
	C/C-C/T	487	1	**0.02**
	T/T	19	0.75 (0.59–0.95)	
	Over-dominant			
	C/C-T/T	366	1	0.12
	C/T	140	0.92 (0.84–1.02)	
	log-Additive			
	0,1,2		0.89 (0.82–0.96)	**0**
**rs662799 relation with VLDL**	**Codominant**			
	C/C	346	1	**0.03**
	C/T	138	0.9 (0.81–1)	
	T/T	18	0.78 (0.61–1)	
	Dominant			
	C/C	346	1	**0.02**
	C/T-T/T	156	0.89 (0.80–0.98)	
	Recessive			
	C/C-C/T	484	1	0.08
	T/T	18	0.80 (0.63–1.03)	
	Over-dominant			
	C/C-T/T	364	1	0.08
	C/T	138	0.91 (0.83–1.01)	
	log-Additive			
	0,1,2		0.9 (0.83–0.97)	**0.01**

*The values represented include the odds ratio, confidence interval and p-values show the mentioned variants to be a significant (p < 0.05) independent predictive factor when controlling for age, sex and BMI for both TG and VLDL levels*.

*TG, triglycerides; VLDL, Very Low-Density Lipoprotein. Significant values (p < 0.05) are highlighted*.

### Haplotype analysis

**Figure 1:**
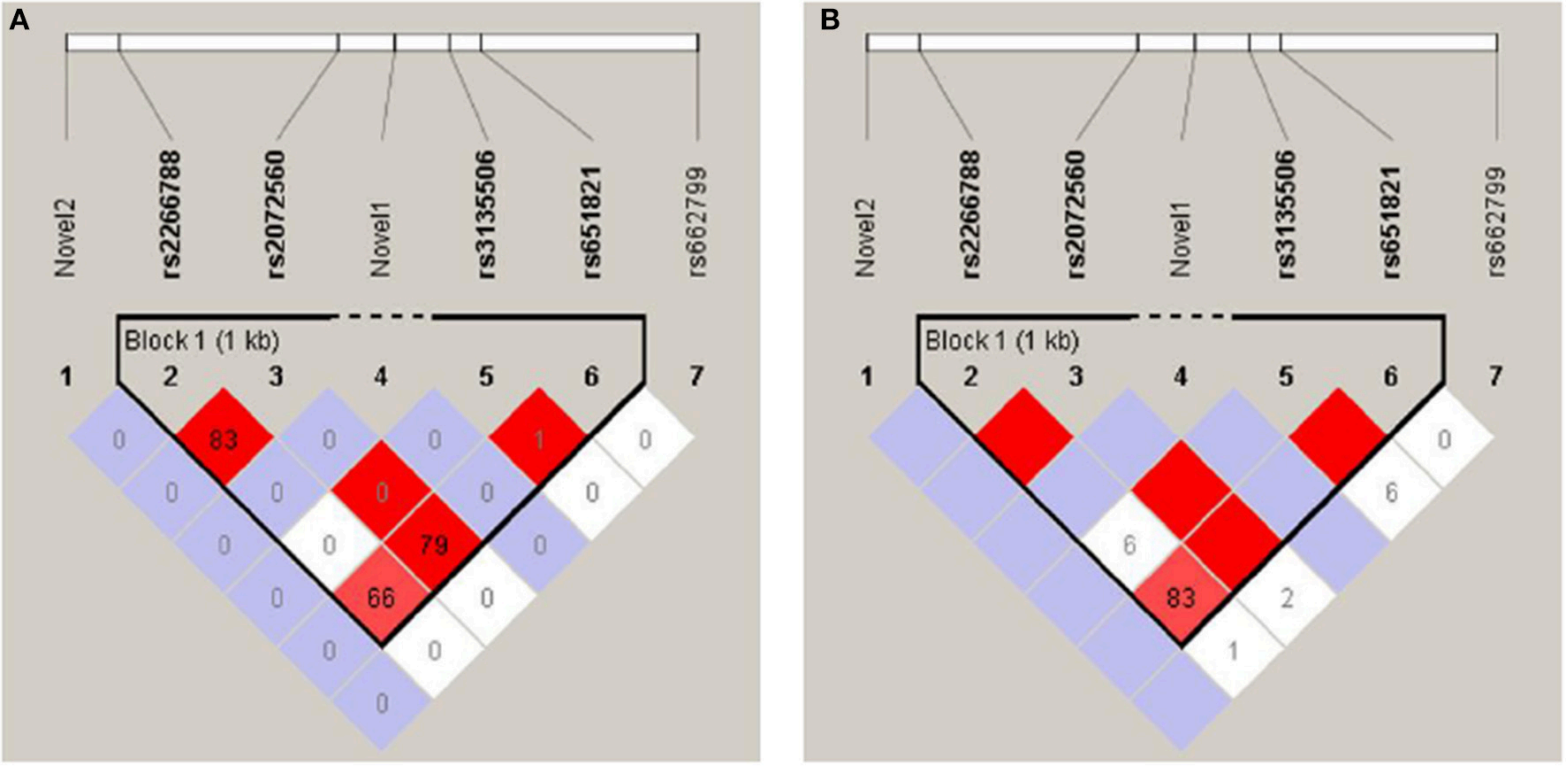
Linkage disequilibrium plots of the studied variants: rs2266788, rs651821, rs2072560, SNP rs3135506, rs662799, novel 1, and novel 2 constructed by Haploview (version 4.2.) **(A)** LD plot representing **r2** values between studied APOA5 SNPs **(B)** LD plot representing **D**′ values between studied APOA5 SNPs.

Linkage disequilibrium (LD) and haplotype analysis between the selected SNPs was expressed in **D**′ and **r**^2^ as presented in Figure 1. Both rs2266788 and rs2072560 SNPs showed disequilibrium (D′ = 1, *r*^2^ = 0.834). In addition, SNPs rs2072560 and rs651821 were also found significantly linked (D′ = 1, *r*^2^ = 0.799). Another strong LD was observed between SNPs re2266788 and rs651821 (D′ = 0.831, *r*^2^ = 0.662). However, rs3135506 SNP showed a perfect D′ value of 1 with SNPs rs2072560 and rs651821 but low r^2^ of 0.008 and 0.01, respectively. On the other hand, five major haplotypes of rs2266788, rs2072560, rs3135506, and rs651821 alleles were identified with TGCA as the common haplotype followed by CACG haplotype in which they represented 79.8 and 11 %, respectively (Table 7). The remaining haplotypes, TGTA, TGCG and CGTA accounted for 4.9, 2.4, and 1.4%, respectively. In total, those five haplotypes accounted for 99.5% of all of the haplotypes in this cohort. Testing the distribution of haplotypes frequencies between high and normal TG level groups indicated no significant association of the resulted haplotypes and TG levels (*P* > 0.05) (Table 7).

**Table 7:** Haplotype groups and frequency of the *APOA5* SNPs among the Kuwaiti Cohort (*n* = 549).

	**rs2266788**	**rs2072560**	**rs3135506**	**rs651821**	**Total haplotype frequency**	**Frequency in samples**		**p-value**
						**High TG**	**Normal TG**	
Haplotype 1	T	G	C	A	0.798 (*n* = 469)	0.12 (*n* = 57)	0.88 (*n* = 412)	0.373
Haplotype 2	C	A	C	G	0.11 (*n* = 143)	0.19 (*n* = 27)	0.81 (*n* = 116)	0.29
Haplotype 3	T	G	T	A	0.049 (*n* = 60)	0.15 (*n* = 9)	0.85 (*n* = 51)	0.956
Haplotype 4	T	G	C	G	0.024 (*n* = 25)	0.2 (*n* = 5)	0.8 (*n* = 20)	0.181
Haplotype 5	C	G	T	A	0.014 (*n* = 20)	0.1 (*n* = 2)	0.9 (*n* = 18)	0.148

^*^p-value < 0.05 significan- TG: Triglycerides.

## Discussion

In several studies, apoA5 displayed substantial impact on lipid levels especially TG leading to the consensus that one of its core functions is TG modulation. Variations in the genetic region encoding this protein has therefore been associated with lipid imbalances among different ethnicities. The present study has re-sequenced the full *APOA5* gene to screen for reported and novel variants and investigate their association with levels of plasma lipids. Re-sequencing the full *APOA5* region in the sampled Kuwaiti Arabs resulted in the identification of 22 genetic variants; 2 novels and 20 previously reported SNPs. Distributions of SNPs were found to be different across the functional component of the *APOA5* gene locus. As expected, the majority of the detected SNPs were found to be in non-coding regions (*n* = 17) than in coding exons (*n* = 5). In 2008, Luis Barreiro and his colleagues hypothesized that non-synonymous mutations and variations within 5′UTR and 3′UTR experience increased selective pressure over intronic and synonymous mutations (Quach et al., 2009). Therefore, results from this study suggest that the Kuwaiti Arabs population is under certain selection pressure. This selection could be toward fixing beneficial *APOA5* alleles that fits better either with their genetic and environmental makeup or toward removing disadvantageous *APOA5* alleles. Most of the detected SNPs in the Kuwaiti Arabs samples were in the 3′UTR (*n* = 7), including a novel SNP. A growing body of evidence has revealed the important role of the 3′UTR in epigenetics as well as regulation of gene expression and that its dysfunction is linked to the pathophysiology of many diseases (Scheper et al., 2007).

SNPs identified in the exonic region revealed three missense variants. One is rs369952307 (c. 1036G>A) SNP that has been recently identified in an African American individual by the group of researchers working on the NHLBI GO ESP (NHLBI Exome Sequencing Project (ESP) 2011). No information about the sample medical condition was provided in that study. In this study, a 22 years old male (BMI = 20.7 Kg/m2) with a normal lipid profile was identified with SNP rs369952307. This sample and the ESP reported sample were identified with a heterozygote genotype. The variant is an outcome of a G to an A transition in exon 4 resulting in an Aspartic (Asp) to Asparagine (Asn) substitution at residue 346 at the C-terminal region of the precursor apoA5 protein. Aspartic acid is acidic, polar and charged amino acid that plays important roles in maintaining the solubility and ionic character of proteins (Patel et al., 2011) unlike asparagine that is neutral, polar and uncharged amino acid. This substitution may therefore affect apoA5 lipid binding ability since this region-terminal has been reported to modulate protein lipid binding activity. To characterize and understand the effect of this variant, a large-scale validation of rs369952307 (c. 1036G>A) SNP among African Americans, Kuwaiti Arabs and other populations is warranted.

The most common identified SNPs were rs36077557 and rs619054 (c.^*^31C>T) with MAF of 31 and 26%, respectively. Those SNPs showed approximately similar allele frequencies like Caucasians and African Americans but diverse from East Asian population (†The International HapMap, 2003; The Genomes Project, 2015). However, both SNPs where not selected for association study as they did not show strong implication in lipid levels in previous studies. Moreover, the rare SNPs identified in the sampled Kuwaiti Arabs are rare among all studied populations in the 1,000 genome project with slightly higher frequencies among Africans. These results may be consistent with the out of Africa theory which states that humans originated in Africa and expanded to Eurasia about one million years ago (Excoffier and Ray, 2008). The bottleneck associated with the expansion may result in those rare SNPs being rarer or lost in non-African populations. These observed interethnic differences in the allelic frequencies between different populations could be due to the distinctive processes of natural selection and adaptation to variable environmental conditions and demographic changes. Consequently, such processes may exert high influence on disease development like dyslipidemia.

Moreover, testing the effect of the commonly studied *APOA5* variants on lipid profile of Kuwaiti Arabs showed controversial results. The functional rs3135506 (S19W) SNP that alters apoA5 signal peptide causing reduced production of the mature thereby affecting apoA5 levels (Olivier et al., 2004; Talmud et al., 2005) have been confirmed to be associated with high TG levels in different populations such as Caucasians (Talmud et al., 2002), African Americans (Klos et al., 2005), and Europeans (De Andrade et al., 2011). In this study however, no association between rs3135506 SNP with TG levels nor with other lipid levels was observed. Yet these findings are consistent with those reported in East Asians (Lai et al., 2003; Li et al., 2011; Yin et al., 2011). Both Kuwaiti Arabs and East Asians showed lower MAF of SNP rs3135506 (0.003, 0.01, respectively) as compared to the populations that reflected the pathological effect of the SNP. This lack of association may be attributed to the ethnicity-specific background and/or the differences in other genetic and environmental factors that may modify the gene effect.

In contrast, the usual association of SNPs rs662799, rs2072560, and rs2266788 minor alleles with elevated TG levels was detected amongst Kuwaiti Arabs (*p* = 0.01, *p* = 0.04, *p* = 0.02, respectively). These findings are comparable with results found in other populations such as Mongolians (Chuluun-Erdene et al., 2017), Caucasians (Talmud et al., 2002), Uyghur population (Li et al., 2014) African Americans (Talmud et al., 2002; Lai et al., 2004), East Asians (Endo et al., 2002; Nabika et al., 2002), and Pakistani population (Shahid et al., 2017). The MAFs of both rs2072560 and rs2266788 in Kuwaiti Arab population was estimated to be 0.8, which resemble frequencies observed between African Americans and Caucasians (Pennacchio et al., 2002). In contrast, rs662799 frequency was extremely low (MAF = 0.04) when compared to other populations such as Chinese (0.3) (Liu et al., 2005), Singaporeans (0.29) (Lai et al., 2003) and Japanese (0.34) (Endo et al., 2002; Nabika et al., 2002). However, it is closer to Caucasians (0.08) (Pennacchio et al., 2001), Hispanic Americans (0.13) (Pennacchio et al., 2002) (Aouizerat et al., 2003) and Tunisians (0.13) (Chaaba et al., 2005). Again, divergence in allelic frequencies between different populations is imputed to different gene-gene or gene-environment interactions among ethnicities.

Moreover, minor alleles of these three SNPs were also found significantly associated with increased VLDL levels in Kuwaiti Arabs. These findings may explain the effect of those variants on apoA5 mechanism of action. Previous studies has explained two mechanisms were apoA5 modulate lipid levels especially TG which are enhancing the catabolism of TG-rich lipoproteins through LPL (Schaap et al., 2004; Merkel et al., 2005) and inhibiting hepatic VLDL, major carrier of TG, assembly and secretion (Weinberg et al., 2003; Schaap et al., 2004). Therefore, the observed elevated TG and VLDL levels associated with SNPs rs662799, rs2072560, and rs2266788 at the *APOA5* locus may reflect the impact of those SNPs on apoA5 mechanism of action. Moreover, a newly discussed evidence on the functionality of the three variants proposed that their rare alleles particularly of rs2266788 create an illegitimate miR-binding site that causes liver posttranscriptional downregulation of *APOA5* by miR-485-4p, a noncoding RNA (miRNA) expressed in the human liver (Caussy et al., 2014). The study suggests that base pairing occurred between APOA5 mRNA carrying the rare alleles and the miR-485-4p leading to mRNA degradation or translation repression (Caussy et al., 2014).

Other studies suggest that the effect of rs2072560 and rs2266788 SNPs is due to their linkage with rs662799 as a leading SNP located in the promoter region. However, linkage analysis results in this study revealed that both SNPs are not linked with rs662799 SNP. The five different haplotypes observed in the Kuwaiti Arab population were very similar to the commonly studied APOA^*^1, APOA^*^2, and APOA^*^3 haplotypes except that haplotypes observed between Kuwaiti Arabs did not involve alleles of rs662799 SNP. In addition, two more haplotypes were observed. The first comprises a mutant allele of rs651821 SNP and the least common includes mutant alleles of both rs2266788 and rs3135506 SNPs. Despite the significant association of some of the detected SNPs with TG levels, the resulted haplotypes frequencies between high and normal TG level patient groups was not significant. These results are different from most of the studies conducted on other populations, but again they may be attributed to the differences in haplotypes frequencies between the different ethnicities as well as other ethnic-specific variations (Li et al., 2014; Sumegi et al., 2017). These findings also indicate the independent role of each variant exerting effect on *APOA5* function or expression rather than in concert.

## Ethics statement

This study has been approved by the Local Ethical Committee at Kuwait University. The sample and phenotypic data collection protocol and informed consents used were in accordance to the year 2000 revised version of the 1975 Helsinki guidelines. Informed consent from each participant in this study was obtained.

## Author contributions

AJ performed all the experiments, analyzed all the data and prepared the manuscript. SA-B prepared the project proposal and study design, supervised the molecular genetic analysis and sample collection and documentation assisted with the data interpretation and preparation of the manuscript. WA-K supervised and assisted with the sequence alignment, its annotations and submission of SNPS and revised the manuscript. AA-S participated in the study design, supervised and assisted with the statistical analysis and revised the manuscript. HA conducted the statistical analysis for the validation and association of the selected variants among the cohort samples. All the authors have read and approved the final manuscript.

### Conflict of interest statement

The authors declare that the research was conducted in the absence of any commercial or financial relationships that could be construed as a potential conflict of interest.

## Supplementary material

Supplementary Table 1 Characteristics and sequences of each primer set (forward and reverse primers) obtained from Primer3 program (version 0.4.0). The length (in base pair) of the overlapping APOA5 gene regions that each set amplifies is also shown.

Supplementary Table 2 Primer sequence and probes for the 7 APOA5 variants genotyped by Taqman Allelic Discrimnation Assay using Real-time PCR.

Supplementary Figure 1 **(A,B)** A chromatograph generated by the sequence at the *APOA5* gene locus showing the novel variants at genomic positions 11:116661525 **(A)** and 11:116660500 **(B)**. The arrow indicates the position of the novel variant which appears as a single peak that was produced by sequencing the target region with the forward primer.

Supplementary Figure 2 **(A–F)** A sample plot from the real-time PCR allelic discrimination assay showing the major allele on the x-axis against the minor allele on the y-axis. Three clusters are shown on each plot representing the three possible genotypes represented by the blue dots for the homozygous wildtype, red dots for the homozygous mutant allele and the green dots for the heterozygous. while near the origin the no template control (NTC) is shown. The genotype assay for each *APOA5* polymorphism is demonstrated as follows: rs2266788 **(A)**, rs651821 **(B)**, rs2072560 **(C)**, SNP rs3135506 **(D)**, rs662799 **(E)**, novel 1 **(F)**.
