# Supplementary material for: Sequence Analysis of APOA5 Among the Kuwaiti Population Identifies Association of rs2072560, rs2266788, and rs662799 With TG and VLDL Levels
Source: Front Genet. 2018 Apr 9;9:112. doi: 10.3389/fgene.2018.00112 (PMC5900548; doi:10.3389/fgene.2018.00112)
Supplement: Supplementary Table 1 — Characteristics and sequences of each primer set (forward and reverse primers) obtained from Primer3 program (version 0.4.0). The length (in base pair) of the overlapping APOA5 gene regions that each set amplifies is also shown. [file Table1.pdf]

Supplementary Table 1. Characteristics and sequences of each primer set (forward and reverse primers) obtained from Primer3 program (version 0.4.0). The length (in base pair) of the overlapping APOA5 gene regions that each set amplifies is also shown.

Sl. No.	Primer set	F/R	Sequence	Tm*	Bp
***APOA5*_1_F**	1	F	5' GAA AGA GCA GCA AGG GAA TG 3'	58.45	526
***APOA5*_1_R**		R	5' AGG CCT GTG CCA CTA CAT CC 3'	58.61	
***APOA5*_2_F**	2	F	5' CCT ATC ACA GCC CAC ACC TT 3'	58.45	637
***APOA5*_2_R**		R	5' TGA CCT GTG GGA AGA CAT CA 3'	58.43	
***APOA5*_3_F**	3	F	5' AGG CCA CTT TCA AGG ACT GA 3'	58.3	502
***APOA5*_3_R**		R	5' GCT CTC CCG GAA GCT CAC 3'	59.45	
***APOA5*_4_F**	4	F	5' GGT CCA GGT TCT GCT GGA T 3'	58.46	659
***APOA5*_4_R**		R	5' CTT GGG GAC AAA GGA GAT GA 3'	58.5	
***APOA5*_5_F**	5	F	5' TTG CTC AAG GCT GTC TTT CA 3'	58.15	579
***APOA5*_5_R**		R	5' GCA GAT CCA TCA GCA GAA GA 3'	57.46	
***APOA5*_6_F**	6	F	5' AGA GCT AGC ACC GCT CCT TT 3'	59.22	663
***APOA5*_6_R**		R	5' ACG CTC CCT GCT ATG TGA CT 3'	58.36	
***APOA5*_7_F**	7	F	5' AGG AGG CTA GGG TGT CTT CC 3'	58.21	522

***APOA5*_7_R**		R	5' CCT GAT ACC AGG GCA CTC AT 3'	58.4	
***APOA5*_8_F**	8	F	5' CCA CGT GGA AGT TCA AAA GAA 3'	58.65	540
***APOA5*_8_R**		R	5' CAT GGG GCA AAT CTC ACT TT 3'	58.43	

*** Tm: annealing temperature**
