# Supplementary material for: Sequence Analysis of APOA5 Among the Kuwaiti Population Identifies Association of rs2072560, rs2266788, and rs662799 With TG and VLDL Levels
Source: Front Genet. 2018 Apr 9;9:112. doi: 10.3389/fgene.2018.00112 (PMC5900548; doi:10.3389/fgene.2018.00112)
Supplement: Supplementary Table 2 — Primer sequence and probes for the 7 APOA5 variants genotyped by Taqman Allelic Discrimnation Assay using Real-time PCR. [file Table2.pdf]

Supplementary Table 2. Primer sequence and probes for the 7 APOA5 variants genotyped by Taqman Allelic Discrimnation Assay using Real-time PCR.

SNP #	Context Sequence
rs651821	AGCACGGCAGCCATGCTTGCCATTA**[C/T]**CTGCTCTGAGAAGACAGGTGGAGGG
rs2072560	TATCCAGGCCGTCAGACTGCTAGCC**[C/T]**CCATCATCTCCTTTGTCCCCAAGTC
rs3135507	TGGGCCTTGGTGTCTTCCCCCACCA**[C/T]**GCGCAACTGCTCCTGCAGCTCCTGC
rs2266788	ATTGGGGAGTCGCAGGAGGCTGGAT**[A/G]**TGCAGGAGACAGCAGCCCCTTTGGT
Novel SNP 1	Novel SNP1_F : ACTAGAGCCAAACTCCAGGATGTA<br>Novel SNP1_R: CAAGCCTGTTTGCCGTGATG
Novel SNP 2	Novel SNP2a_F: TGCTCCTGCAGCTCCTG<br>Novel SNP2a_R: AGCCCTACACGATGGATCTGA
