# Supplementary material for: Sequence Analysis of APOA5 Among the Kuwaiti Population Identifies Association of rs2072560, rs2266788, and rs662799 With TG and VLDL Levels
Source: Front Genet. 2018 Apr 9;9:112. doi: 10.3389/fgene.2018.00112 (PMC5900548; doi:10.3389/fgene.2018.00112)
Supplement: Supplementary Figure 1 — (A,B) A chromatograph generated by the sequence at the APOA5 gene locus showing the novel variants at genomic positions 11:116661525 (A) and 11:116660500 (B). The arrow indicates the position of the novel variant which appears as a single peak that was produced by sequencing the target region with the forward primer. [file Image1.PDF]

A.

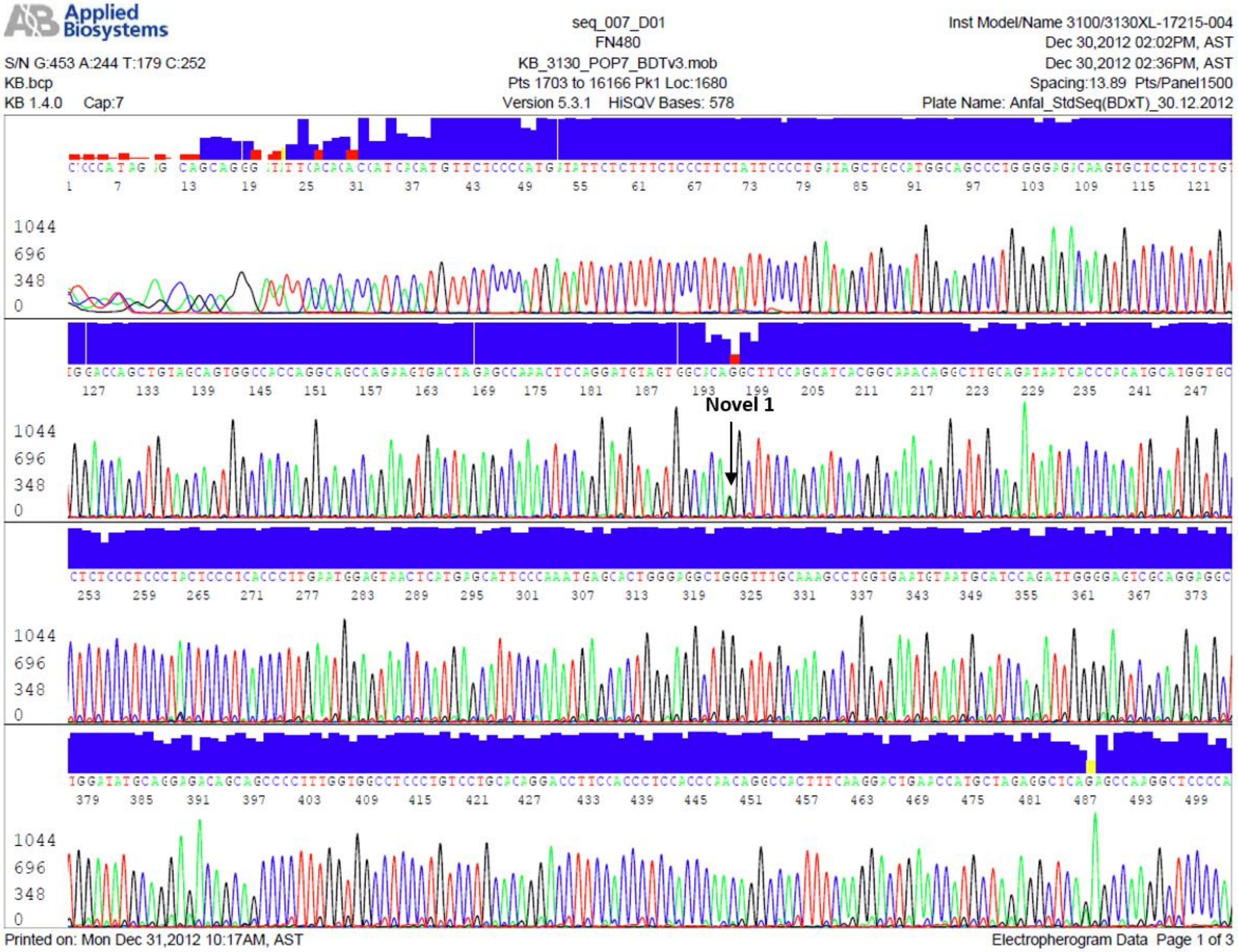

Applied Biosystems
seq_007_D01
FN480
KB_3130_POP7_BDTv3.mob
Pts 1703 to 16166 Pk1 Loc:1680
Version 5.3.1 HiSQV Bases: 578
Inst Model/Name 3100/3130XL-17215-004
Dec 30,2012 02:02PM, AST
Dec 30,2012 02:36PM, AST
Spacing:13.89 Pts/Panel1500
Plate Name: Anfal_StdSeq(BDxT)_30.12.2012
S/N G:453 A:244 T:179 C:252
KB.bcp
KB 1.4.0 Cap:7
Novel 1
Printed on: Mon Dec 31,2012 10:17AM, AST
Electropherogram Data Page 1 of 3

B.

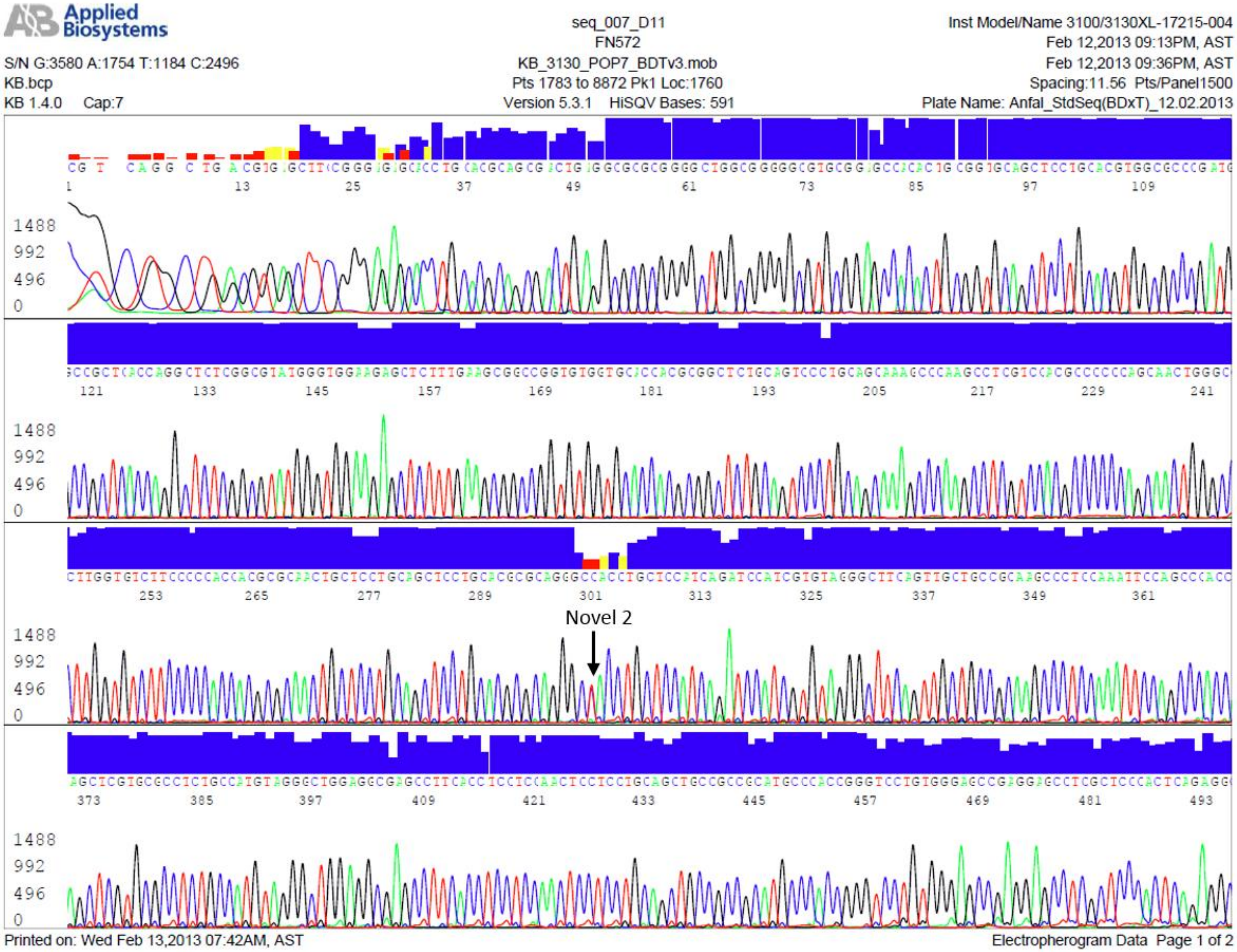
Applied Biosystems
seq_007_D11
FN572
KB_3130_POP7_BDTv3.mob
Pts 1783 to 8872 Pk1 Loc:1760
Version 5.3.1 HiSQV Bases: 591
Inst Model/Name 3100/3130XL-17215-004
Feb 12,2013 09:13PM, AST
Feb 12,2013 09:36PM, AST
Spacing:11.56 Pts/Panel1500
Plate Name: Anfal_StdSeq(BDxT)_12.02.2013
S/N G:3580 A:1754 T:1184 C:2496
KB.bcp
KB 1.4.0 Cap:7
1488
992
496
0
Novel 2
Printed on: Wed Feb 13,2013 07:42AM, AST
Electropherogram Data Page 1 of 2
