# Supplementary figures and images for: Sequence Analysis of APOA5 Among the Kuwaiti Population Identifies Association of rs2072560, rs2266788, and rs662799 With TG and VLDL Levels
Source: Front Genet. 2018 Apr 9;9:112. doi: 10.3389/fgene.2018.00112 (PMC5900548; doi:10.3389/fgene.2018.00112)

A.

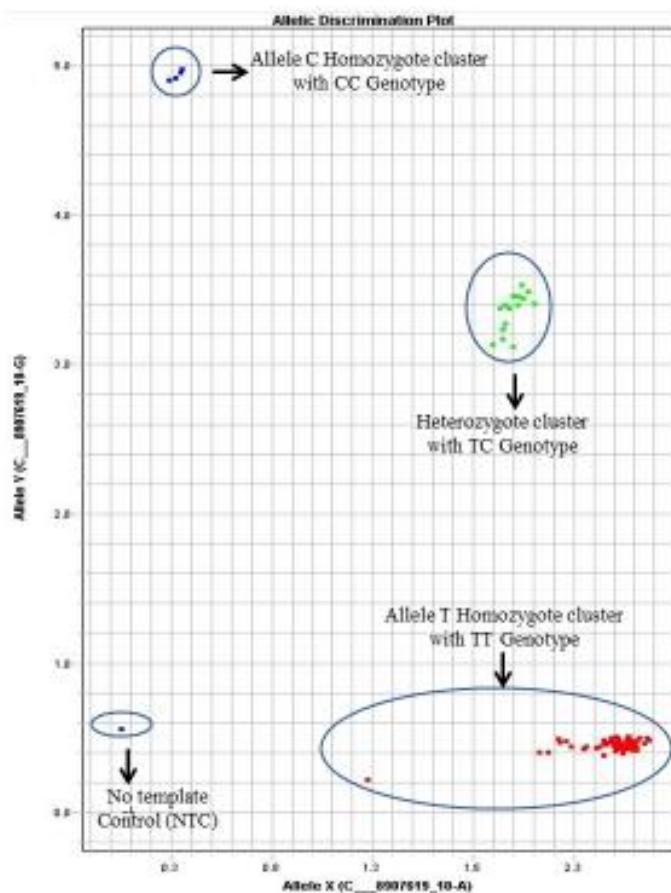

B.

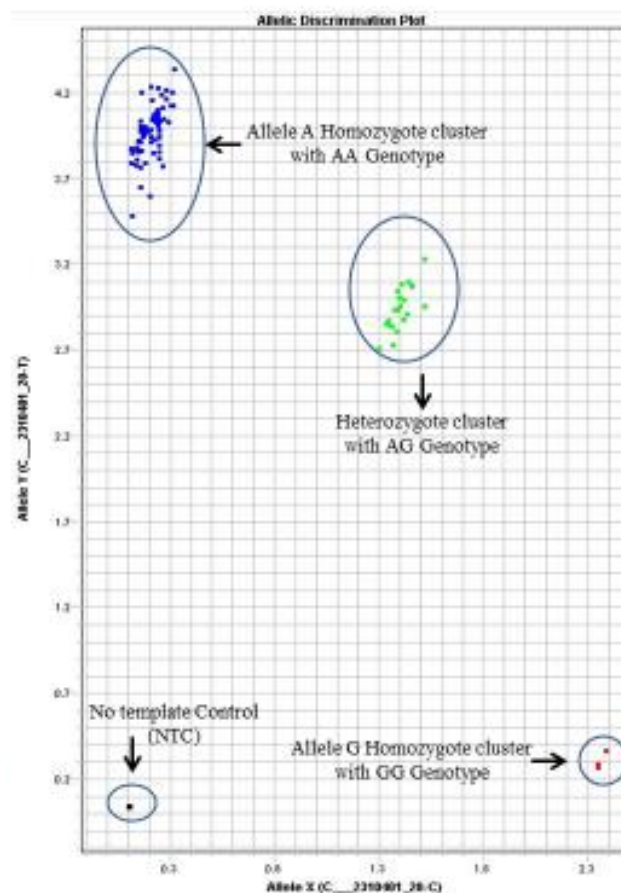

C.

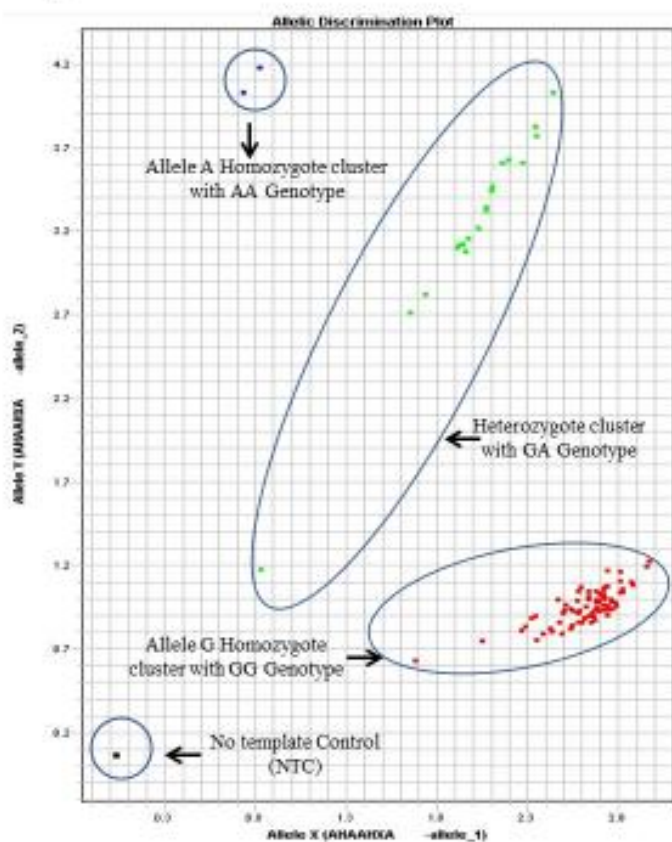

D.

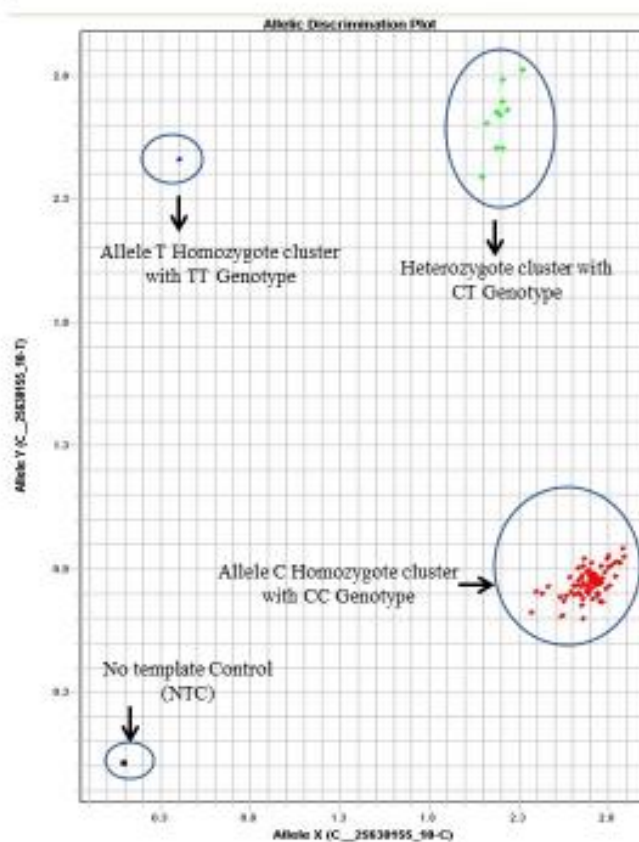

E.

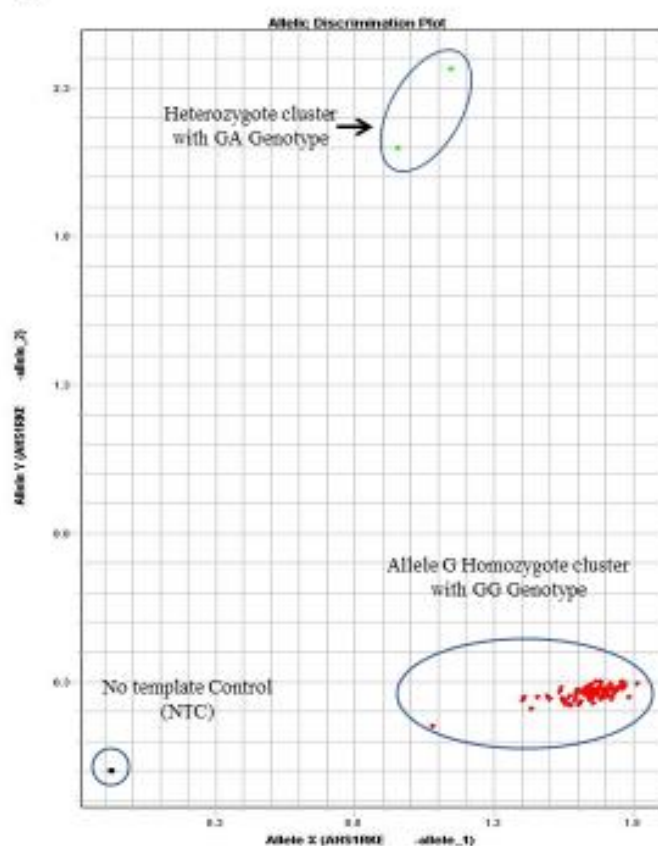

F.

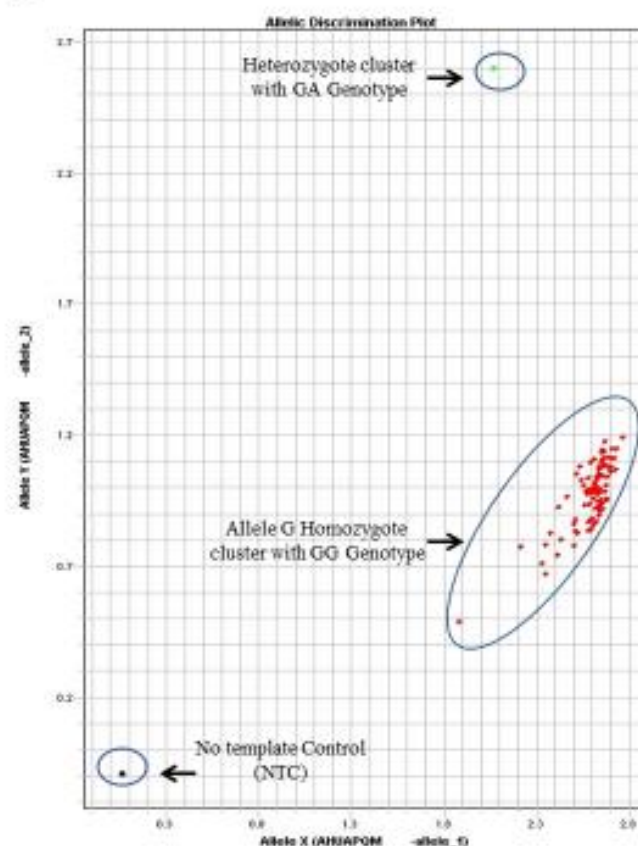

Supplement: Supplementary Figure 2 — (A–F) A sample plot from the real-time PCR allelic discrimination assay showing the major allele on the x-axis against the minor allele on the y-axis. Three clusters are shown on each plot representing the three possible genotypes represented by the blue dots for the homozygous wildtype, red dots for the homozygous mutant allele and the green dots for the heterozygous. while near the origin the no template control (NTC) is shown. The genotype assay for each APOA5 polymorphism is demonstrated as follows: rs2266788 (A), rs651821 (B), rs2072560 (C), SNP rs3135506 (D), rs662799 (E), novel 1 (F). [file Image2.PDF]
